# Sonic Hedgehog Signaling Drives Proliferation of Synoviocytes in Rheumatoid Arthritis: A Possible Novel Therapeutic Target

**DOI:** 10.1155/2014/401903

**Published:** 2014-03-06

**Authors:** Mingxia Wang, Shangling Zhu, Weixiang Peng, Qiuxia Li, Zhaoxia Li, Minqi Luo, Xiaoxue Feng, Zhuofeng Lin, Jianlin Huang

**Affiliations:** ^1^School of Pharmaceutical Sciences, Wenzhou Medical University, Wenzhou, Zhejiang 325035, China; ^2^Department of Rheumatology, The Third Affiliated Hospital of Sun Yat-Sen University, Guangzhou 510630, China; ^3^Department of Rheumatology of Zhongshan Affiliated Hospital, Sun Yat-Sen University, Zhongshan, Guangdong 528400, China

## Abstract

Sonic hedgehog (Shh) signaling controls many aspects of human development, regulates cell growth and differentiation in adult tissues, and is activated in a number of malignancies. Rheumatoid arthritis (RA) is characterized by chronic synovitis and pannus formation associated with activation of fibroblast-like synoviocytes (FLS). We investigated whether Shh signaling plays a role in the proliferation of FLS in RA. Expression of Shh signaling related components (Shh, Ptch1, Smo, and Gli1) in RA synovial tissues was examined by immunohistochemistry (IHC) and in FLS by IHC, immunofluorescence (IF), quantitative RT-PCR, and western blotting. Expression of Shh, Smo, and Gli1 in RA synovial tissue was higher than that in control tissue (*P* < 0.05). Cyclopamine (a specific inhibitor of Shh signaling) decreased mRNA expression of Shh, Ptch1, Smo, and Gli1 in cultured RA FLS, Shh, and Smo protein expression, and significantly decreased FLS proliferation. Flow cytometry analysis suggested that cyclopamine treatment resulted in cell cycle arrest of FLS in G_1_ phase. Our data show that Shh signaling is activated in synovium of RA patients *in vivo* and in cultured FLS form RA patients *in vitro*, suggesting a role in the proliferation of FLS in RA. It may therefore be a novel therapeutic target in RA.

## 1. Introduction

Rheumatoid arthritis (RA) is characterized by persistent synovitis, systemic inflammation, and autoantibodies (particularly rheumatoid factor and anticitrullinated peptide) [1]. The dominant local cell populations in joints affected by rheumatoid arthritis are synovial and cartilage cells. Synovial cells can be divided into two types, fibroblast-like and macrophage-like synoviocytes. Fibroblast-like synoviocytes (FLS) show abnormal behaviour in RA. In experimental models, coimplantation of FLS with cartilage leads to fibroblasts invading cartilage [2], which is correlated with joint destruction [3].

FLS contribute significantly to the perpetuation of disease and perhaps also play a role in the initiation phase. These FLS from RA patients constitute a quite unique cell type and they distinguish RA from other inflammatory conditions of the joints. A number of studies have demonstrated that FLS from RA patients showed alterations in morphology and behaviour, including molecular changes in signaling cascades, in apoptosis, expression of adhesion molecules, and matrix-degrading enzymes. These changes appear to reflect a stable activation of FLS in RA, which occurs independently through continuous exogenous stimulation. As a result, FLS are no longer considered passive bystanders in RA but active players in the complex intercellular network of RA [4].

Current evidence indicates that FLS in rheumatoid synovium are one of the principal cells involved in pannus formation, and they are key players in the destruction of cartilage and bone in the joint. These FLS have been shown to proliferate in an anchorage-independent manner, lack contact inhibition, and constitutively express cytokines, oncogenes, and cell cycle proteins, which is suggestive of transformation [5].

Sonic hedgehog (Shh) is a member of Hedgehog (Hh) family of proteins, acts as a morphogen in embryonic development [6]. In the absence of Shh ligand, its receptor patched (Ptch) 1, a twelve-transmembrane protein, exerts an inhibitory effect on Smoothened (Smo), a seven-pass transmembrane receptor related to the G protein coupled receptor (GPCR) family, and thereby attenuates downstream signal transduction. When the ligand binds to Ptch1, the repression of Smo is relieved, resulting in the activation of transcription factors Gli1-3 and consequentially in expression of a panel of downstream target genes [7]. Stimulation of hedgehog signaling induces carcinogenesis or promotes cell survival in cancers of multiple organs. Aberrant activation of Shh signaling has been reported in various cancers, including basal cell carcinoma, medulloblastoma, pancreatic cancer, and gastric cancer, either by directly regulating cellular growth and survival [8] or by indirectly driving carcinoma cell growth by influencing the tumor stroma [9, 10].

Whether the Shh signaling pathway is activated in synovial tissue in active RA, and if it plays a role in proliferation of FLS in RA, is still unknown. Therefore, expression of Shh signaling pathway related components in synovial tissues from patients with RA and the effect of cyclopamine (a specific inhibitor of Shh signaling) on expression of Shh signaling pathway related components and cell proliferation of cultured FLS from RA patients were observed in this study.

## 2. Materials and Methods

### 2.1. Patients, Controls, and Ethics Statement

Han Chinese subjects, including 10 patients with active RA (*n* = 10, 4 males, 6 females, mean age 50.4 ± 11.3), and 5 age-matched control patients with knee trauma (*n* = 5, 3 males, 2 females, mean age 49.7 ± 12.2) were recruited from the Third Affiliated Hospital of Sun Yat-sen University. Synovial tissues were obtained when the patients were undergoing knee arthroscopy. RA patients satisfied the American College of Rheumatology (ACR) 1987 revised classification criteria for RA [11], with disease activity (Disease Activity Score using 28 joint counts) >3.2 [12]. The study was approved by the Ethics Committee of the Third Affiliated Hospital of Sun Yat-sen University. Written informed consent was obtained from all patients.

Clinical data were available for all samples in the RA group, including rheumatoid factor (RF) (176.3 ± 118.4) IU/mL, anti-CCP antibody (30.5 ± 8.5) U/mL, and term of disease (12.8 ± 4.3) years. RF and anti-CCP antibodies were all three times the normal level.

### 2.2. Immunohistochemistry

Synovial tissues from patients with RA and controls were fixed in 4% paraformaldehyde solution, embedded in paraffin blocks, and cut into 4**μ**m thick sections. Slides were preincubated in 3% H_2_O_2_ for 5 minutes to block endogenous peroxidase. Nonspecific binding was blocked with 10% goat serum for 30 minutes and sections were subsequently incubated with a mouse anti-human Shh monoclonal antibody (1 : 100 dilution, Sigma, USA), goat anti-human Ptch1 polyclonal antibody (1 : 80 dilution, Santa Cruz, INC, USA), rabbit anti-human Gli1 polyclonal antibody (1 : 25 dilution, Santa Cruz, INC, USA), or rabbit anti-human Smo polyclonal antibody (1 : 100 dilution, Abcam, UK), respectively, in a moist chamber at 4°C overnight. After washing with phosphate buffered saline (PBS), sections were then incubated with horseradish peroxidase- (HRP-) conjugated goat anti-rabbit or rabbit anti-goat or goat anti-mouse IgG (Dako Denmark A/S, Glostrup Denmark) for 1 hour at room temperature. Color was developed using diaminobenzidine (DAB), followed by counterstaining with hematoxylin. Negative controls were carried out by omission of primary antibodies.

Staining scores were calculated by semiquantitative optical analysis. Cell membranes, cytoplasm, and/or nuclei that contained yellow or brown granules were considered positively stained cells. Five fields per slide at high magnification (400 times) were evaluated by fluorescence microscopy, the number of positive cells and the total number of cells were determined, and the positive expression rates were calculated. The positive expression rate (%) was defined as (number of positive cells/total cells) × 100%. Negative expression (−) indicates that no positive cells were found; weakly positive expression (+) indicates a positive expression rate of <25%; moderately positive expression (++) indicates a positive expression rate of 25% to 75%; strongly positive expression (+++) indicates a positive expression rate of >75% [13]. Immunostaining was evaluated by two independent observers (XC and AH) who were blinded for the patients' diagnosis, clinical and pathological features.

### 2.3. Cell Culture

FLS for tissue culture from RA patients were isolated from synovial tissues as described previously [14]. Briefly, the collected synovial tissues were minced and cultured as explant pieces in a flask with DMEM (Hyclone, USA) supplemented with 10% fetal bovine serum (FBS) (Hyclone, USA). Within 14 days, FLS migrated out from the tissue explants and formed confluent monolayers. The cells were collected by trypsinization and reseeded into flasks for expansion. FLS from passages 3 to 5 were used for each experiment after being identified by morphology and purity analysis. All cells were incubated at 37.0°C in a humidified atmosphere containing 5% CO_2_.

### 2.4. Cell Immunofluorescence

Cultured RA FLS from passages 3 to 5 were plated in laser confocal Petri dishes at a density of 1 × 10^4^/mL in DMEM supplemented with 10% FBS, and serum-starved for 12 hours before experiments. The expression of Shh signaling pathway related components (including Shh, Ptch1, Gli1, and Smo) in FLS was observed using laser confocal microscopy (Zeiss LSM 710) or fluorescence microscopy according to the manufacturer's instructions. Briefly, FLS were fixed in 4% paraformaldehyde on cell culture Petri dishes for 5 minutes and treated in a mixture of Tris-buffered saline and Tween-20 (TBST) twice for 7 minutes. After permeabilization with 0.25% Triton X-100 for 5 minutes and incubation in blocking solution (5% goat serum in Tris-buffered saline plus 0.1% Tween-20) for 1 hour, cells were stained with anti-Shh antibody (1 : 200 dilution), anti-Ptch1 antibody (1 : 100 dilution), anti-Gli1 antibody (1 : 100 dilution), or anti-Smo antibody (1 : 300 dilution) in a moist chamber at 4°C overnight. The staining was performed using a secondary Alexa Fluor conjugated antibody (goat anti-rabbit, rabbit anti-goat, or goat anti-mouse, Invitrogen, USA), using a 1 hour incubation.

### 2.5. RNA Isolation and Real-Time PCR Analysis

Total RNA was extracted from cultured RA FLS using Trizol reagent (Invitrogen, CA, USA) according to the manufacturer's protocol. RNA concentration and purity were assessed by measuring optical density at 260/280 nm.

cDNA was synthesized from total RNA using the PrimeScript RT reagent Kit (Takara, Biotechnology, Dalian, China). The following reagents were added: 5x PrimeScript Buffer (2.0**μ**L), RT Enzyme Mix (0.5**μ**L), total RNA (1**μ**g), oligo (dT) primer (0.5**μ**L), and random 6-mers (0.5**μ**L), and the reaction volume was brought to 20**μ**L with RNase free water. Then the reagents were mixed gently and centrifuged briefly. Finally, this mixture was incubated at 37°C for 15 minutes for initiation and was terminated by heating at 85°C for 5 seconds.

After reverse transcription, cDNA was used for real-time PCR to quantify Shh, Ptch1, Gli1, and Smo mRNA expression levels relative to GAPDH using the 2^−ΔΔCT^ method. Relative expression ratios were expressed as fold changes in mRNA levels compared to control group levels. Real-time PCR components were as follows: cDNA (2**μ**L), primer (0.4**μ**L each), SYBR* Premix Ex Taq* (10**μ**L), ROX Reference Dye II (0.4**μ**L), and dH_2_O (6.8**μ**L). The primers for amplification were as follows (forward, reverse): for Shh (5′-TCCAGAAACTCCGAGCGATTTAAG-3′, 5′-CACTCCTGGCCACTGGTTCA-3′); Ptch1 (5′-CTGCGTCAGCAGAGTGATTC-3′, 5′-AGCTGAGGGTGTCCTGTGTC-3′); Gli1 (5′-AGGGAGTGCAGCCAATACAG-3′, 5′-CCGGAGTTGATGTAGCTGGT-3′); Smo (5′-GCCATGTTTGGAACTGGCATC-3′, 5′-ATCCGCTTTGGCTCATCGTC-3′); GAPDH (5′-GCACCGTCAAGGCTGAGAAC-3′, 5′-TGGTGAAGACGCCAGTGGA-3′). The real-time quantitative PCR assay was performed using SYBR* Premix Ex Taq* (Takara, Biotechnology, Dalian, China) on an ABI-7500 Thermal Cycler (Applied Biosystem, USA) in 96-well optical reaction plates in triplicate. The protocol for all genes was template predenaturation (30 seconds at 95°C), PCR reaction (5 seconds at 95°C and 34 seconds at 60°C) for 40 cycles. A melting curve analysis was performed at the end of the amplification process according to the manufacturer's protocol to confirm specificity of amplification. Each reaction included positive and negative controls.

### 2.6. Western Blot Analysis

Western blot analyses were performed utilizing standard procedures. Briefly, total proteins were extracted using lysis buffer (Cell Signal Technology, MA, USA) following the manufacturer's instructions. 35**μ**g proteins were incubated at 100°C for 5 minutes and then loaded and resolved using 8% sodium dodecyl sulfate-polyacrylamide gel electrophoresis (SDS-PAGE). The separated proteins were then transferred to a polyvinylidene fluoride (PVDF) membrane. Membranes were blocked at room temperature for 2 hours (3% bovine serum albumin in Tris-buffered saline plus 0.1% Tween-20) and incubated overnight at 4°C with primary antibodies in blocking solution. Primary antibodies were as follows: anti-Shh antibody (1 : 1000 dilution) and anti-Smo antibody (1 : 2500 dilution). Subsequently membranes were incubated for 1 hour at room temperature with HRP-conjugated secondary antibodies. Finally, protein signals were detected by chemiluminescence (FUJIFILM, Shanghai, China) using enhanced chemiluminescence (ECL) detection reagents (EMD Millipore Corporation, MA, USA). Densitometry was performed using Quantity One software (Bio-Rad Laboratories, CA, USA). The expression of GAPDH was used as an internal standard.

### 2.7. Cell Viability Assays

Cell viability and proliferation were assessed using Cell Counting Kit-8 (CCK-8, Dojindo, Tokyo, Japan) according to the manufacturer's instructions. Briefly, cultured RA FLS were plated in 96-well plates at a density of 2.5 × 10^3^/mL in DMEM supplemented with 10% FBS. Cells were serum-starved for 12 hours and incubated with cyclopamine (LC Laboratories, USA) (10**μ**mol/L) for another 48 hours. Cyclopamine was dissolved at 20 mg/mL in 95% ethanol and the solutions were diluted to the final concentration with 10% FBS. After the incubation, 10**μ**L of the CCK-8 solution was added to each well of the plate, which was then further incubated for 4 hours. The absorbance at 450 nm was measured using a microplate reader. The amount of the formazan dye generated by dehydrogenases in cells is directly proportional to the number of living cells.

### 2.8. Flow Cytometry

Cultured FLS from RA were seeded in 6-well plates at a density of 1 × 10^5^/mL for 24 hours. Cells were serum-starved for 12 hours, followed by incubation with cyclopamine (10**μ**mol/L) for 48 hours. Cell cycle phases were determined by flow cytometry (propidium iodide (PI) method). Briefly, cells were collected, washed twice with cold PBS, centrifuged, and fixed in 1 mL 75% ethanol (−20°C precooled) overnight at −20°C. Fixed cells were washed twice in PBS (pH 7.4) and incubated with 20**μ**L RNase A (100**μ**g/mL) (Invitrogen, CA, USA) for 30 minutes at 37°C. Nuclei were stained with PI (50**μ**g/mL) (Sigma-Aldrich, MO, USA) in the dark for 30 minutes at 4°C. Then the stained cells were analyzed using a FACSCalibur flow cytometer (Becton Dickinson, USA), measuring the fluorescence emission at 630 nm using 488 nm excitation. For each analysis, 10,000 events were evaluated.

### 2.9. Statistical Analysis

SPSS version 17.0 was used for all statistical analyses. Values are presented as means ± standard deviation (S.D.). Comparisons of numerical data between groups were performed by independent-samples *t*-test. Statistical significance was set at *P* < 0.05.

## 3. Results

### 3.1. Shh, Ptch1, Smo, and Gli1 Protein Were Highly Expressed in Synovium from Patients with RA

**Figure 1:**
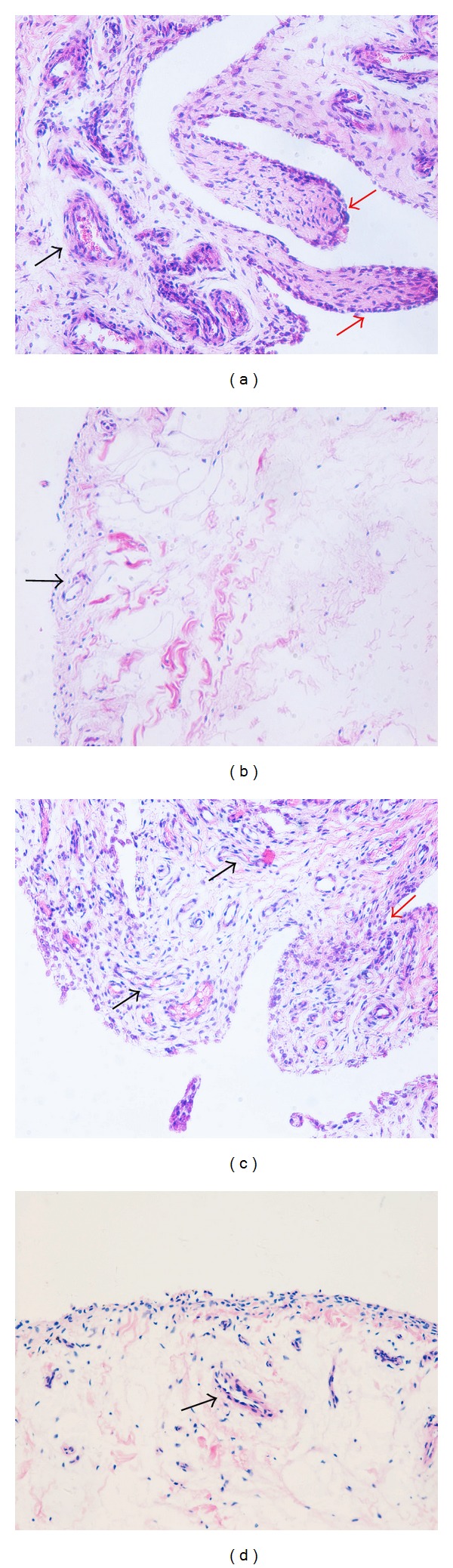
Hematoxylin eosin staining for synovial tissue (original magnification ×200). Hematoxylin eosin staining distinguished synovium of RA patients from synovium of controls. Inflammatory cell infiltration, synovial cell proliferation, and pannus formation are visible in synovium of RA patients ((a), (c)). Fewer inflammatory changes are detectable in synovium from knee trauma patients ((b), (d)). Red arrow: synovial cell proliferation. Black arrow: pannus formation.

Inflammation of synovial tissue was observed by hematoxylin-eosin staining of specimens from 10 patients with RA and 5 patients with knee trauma as control group. Pannus abundance was calculated at a magnification of 200 times. Histology showed typical synovitis in RA specimens, including inflammatory cell infiltration, synovial cell proliferation, and pannus formation (Figures 1(a) and 1(c)). However, few signs of inflammation and neither synovial cell proliferation nor pannus formation were observed in controls (Figures 1(b) and 1(d)).

**Figure 2:**
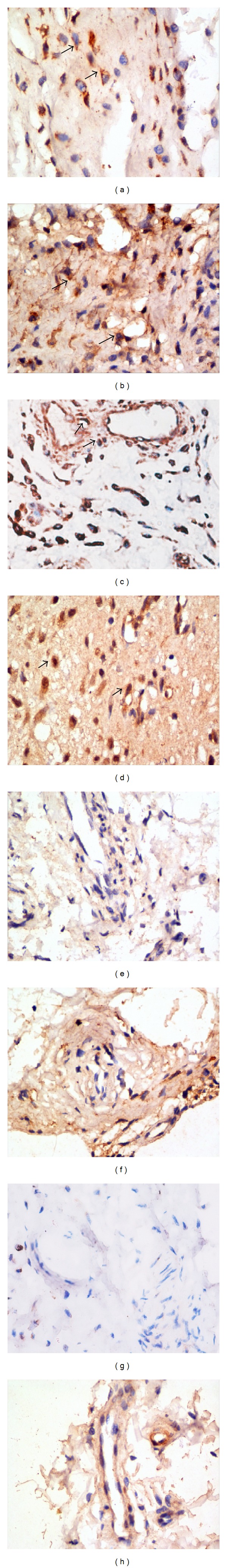
Shh, Ptch1, Smo, and Gli1 were highly expressed in RA synovial tissue (original magnification ×400). Immunohistochemistry demonstrated that Shh, Ptch1, Smo, and Gli1 were expressed in synovium from RA patients ((a), (b), (c), (d)). Localized expression of Shh was observed at the cell membrane and in the cytoplasm of synoviocytes, mainly at the cell membrane (a); expression of Ptch1 and Smo was observed in the plasma of synoviocytes ((b), (c)). Gli1 was expressed mainly in the nucleus of synoviocytes (d). Shh, Ptch1, Smo, and Gli1 were expressed in synovium from patients with knee trauma in a similar pattern, but their expression levels were relatively low compared to those in RA synovium. (a), (b), (c), and (d) were RA specimens; (e), (f), (g), and (h) were controls. (a) and (e): Shh; (b) and (f): Ptch1; (c) and (g): Smo; (d) and (h): Gli1. Shh, Sonic hedgehog; Ptch1, patched 1; Smo, smoothened; Gli1, glioma-associated oncogene 1.

Immunohistochemistry showed that Shh, Ptch1, Smo, and Gli1 were expressed at high levels in synovium from RA patients (Figures 2(a), 2(b), 2(c), and 2(d)). Localized expression of Shh was observed at the cell membrane and cytoplasm of synoviocytes (mainly expressed at the cell membrane, Figure 2(a)). Expression of Ptch1 and Smo was observed in the plasma of synoviocytes (Figures 2(b) and 2(c)). Gli1 was expressed mainly in the nucleus of synoviocytes (Figure 2(d)). Shh, Ptch1, Smo, and Gli1 were expressed in synovium from patients with knee trauma in a similar pattern, but their expression levels were relatively low compared to those in synovium from RA patients.

**Figure 3:**
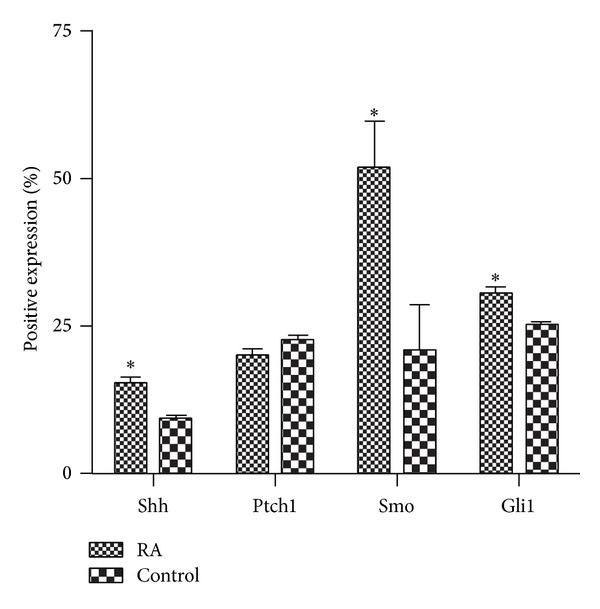
Positive expression rates of Shh-related proteins in synovium from RA patients and controls. The positive expression rate of Shh, Smo, and Gli1 protein in synovium of RA patients was higher than that in the control group, while there was no difference in the expression of Ptch1. *P < 0.05 versus control. The positive expression rate is defined as (number of positive cells/total cells) × 100%.

The results of a semiquantitative analysis of expression of Shh, Ptch1, Smo, and Gli1 in synovium of RA patients and controls are shown in Table 1. Results showed that the rate of moderately positive expression of Shh, Smo, and Gli1 in the RA group (80%, 60%, and 20%, resp.) was higher than that in the control group (40%, 0%, and 0%, resp.). The rate of strongly positive expression of Smo protein in the RA group was 40%. However, strongly positive expression of Shh, Ptch1, and Gli1 was not found in either group. We also performed a statistical analysis of the positive expression rates in the RA and control group. Results showed that Shh, Smo, and Gli1 protein expression in the RA group was higher than that in the control group, while there was no difference in the expression of Ptch1 (*P* < 0.05) (Figure 3).

### 3.2. Shh, Ptch1, Smo, and Gli1 Proteins Were Expressed in Cultured FLS from RA Patients

**Figure 4:**
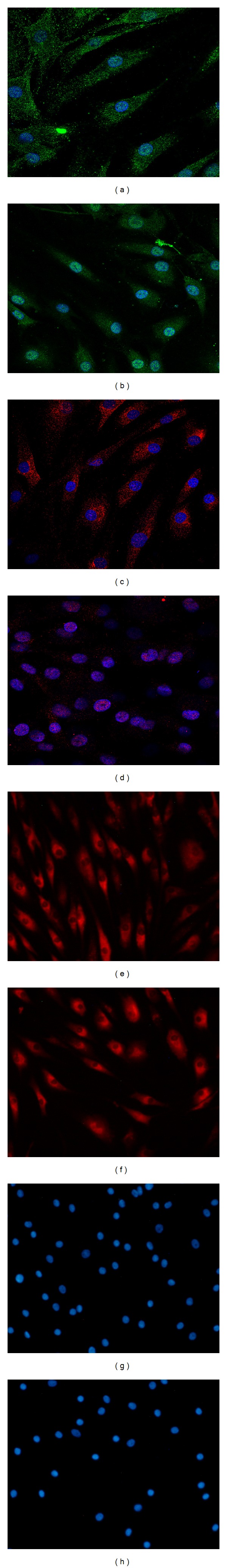
Detection of protein expression of Shh signaling pathway related components in cultured FLS from RA patients by cell immunofluorescence assay (×200). Expression of Shh (a), Ptch1 (b), Smo (c), and Gli1 (d) protein was observed using laser confocal microscopy. Smo was assayed by fluorescence microscopy ((e), (f)). After treatment with cyclopamine (10**μ**mol/L) for 48 hours, there were considerably fewer cells expressing Smo (f) compared to the control group (e). (g) and (h) show a nuclear stain of the same field as (e) and (f).

Expression of Shh, Ptch1, Smo, and Gli1 protein in cultured RA FLS was determined using laser confocal microscopy and fluorescence microscopy. Cultured RA FLS expressed the above proteins in a similar pattern as synoviocytes in sections of synovium from RA patients, indicating that Shh signaling was activated in cultured RA FLS. Localized expression of Shh was observed in the cytoplasm of synoviocytes (Figure 4(a)), and expression of Ptch1 (Figure 4(b)) and Smo (Figure 4(c)) was also observed in the cytoplasm of synoviocytes. Gli1 was expressed mainly in the nucleus of synoviocytes (Figure 4(d)). After incubation with cyclopamine (10**μ**mol/L) for 48 hours, there were considerably fewer cells expressing Smo (Figure 4(f)) compared to untreated controls (Figure 4(e)).

### 3.3. Cyclopamine Decreased Expression of Shh, Ptch1, Smo, and Gli1 mRNA in Cultured FLS from RA Patients

**Figure 5:**
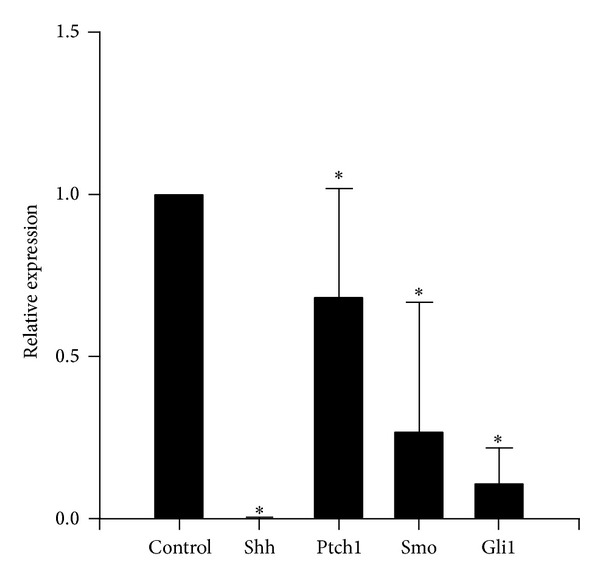
Cyclopamine decreased expression of Shh, Ptch1, Smo, and Gli1 mRNA in cultured FLS from RA. Cells were stimulated, treated, and harvested as described in Section 2. cDNA was synthesized and used for quantitative PCR. Relative quantification of gene expression was performed by the 2^−ΔΔCt^ method. Means ± S.D. of 3 independent experiments are shown. *P < 0.05 versus control. Control group: FLS from RA patients were cultured in DMEM supplemented with 10% FBS. Differences between means of two groups were performed by independent-samples *t*-test.

Using real-time PCR, we examined the effect of cyclopamine on the mRNA expression of Shh signaling pathway related components in FLS. Relative quantification of gene expression was performed by the 2^−ΔΔCt^ method. Results showed that the expression of Shh, Ptch1, Smo, and Gli1 mRNA in the cyclopamine group (0.002 ± 0.012, 0.68 ± 0.34, 0.11 ± 0.11, and 0.27 ± 0.40, resp.) was lower than that in the control group (*P* < 0.05) (Figure 5).

### 3.4. Cyclopamine Decreased Expression of Shh and Smo Proteins in Cultured FLS from RA Patients

**Figure 6:**
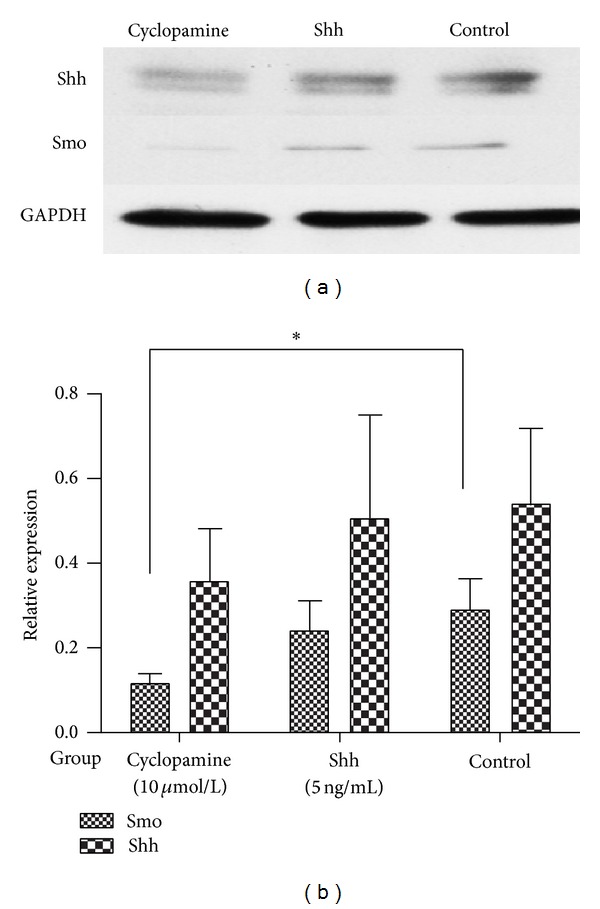
Cyclopamine decreased Shh and Smo protein expression in cultured RA FLS. Cells were stimulated, treated, and harvested as described in Section 2. Means ± S.D. of 3 independent experiments are shown. Expression of Smo and Shh was significantly lower in cyclopamine-treated FLS than in the control group (*P* < 0.05). There was no significant difference in Smo and Shh protein expression between Shh stimulated FLS and control cells. *P < 0.05 versus control group.

Based on the findings above, the effect of cyclopamine on Shh and Smo protein expression in cultured FLS from RA patients was also investigated. Results suggested that the expression of Smo and Shh was lower in the cyclopamine-treated group than that in the control group (Figure 6, *P* < 0.05). We also observed protein expression of Shh and Smo in cultured RA FLS incubated with Shh (5**μ**g/L) for 48 hours. However, there was no significant difference in Shh and Smo protein expression between the Shh stimulated group and the control group (Figure 6, *P* > 0.05).

### 3.5. Cyclopamine Decreased the Proliferation of Cultured FLS Cells

**Figure 7:**
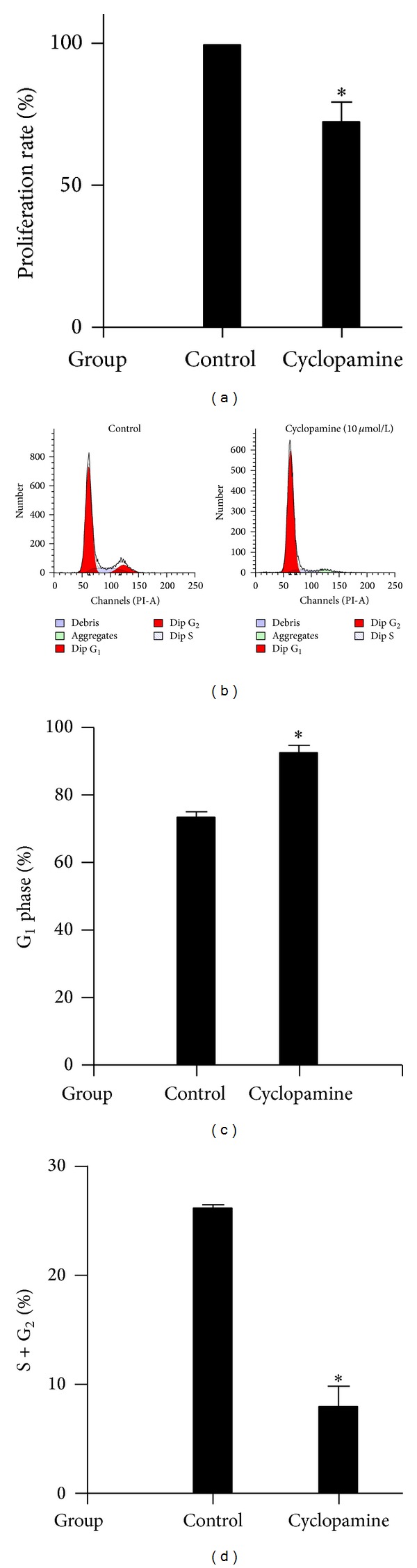
Cyclopamine decreased the viability of cultured FLS from RA patients. Cells were stimulated, treated, and harvested as described in Section 2. (a) Cell proliferation was determined using the Cell Counting Kit-8. The cell proliferation rate in cyclopamine-treated FLS was significantly lower than that in the control group (100 ± 0)% (*P < 0.05) ((b), (c)). The proportion of cells in the G_1_ phase was significantly higher in cyclopamine-treated FLS than in controls, indicating cell cycle arrest (*P < 0.05). (d) In cyclopamine-treated FLS the percentage of cells in the S + G_2_ phase was significantly reduced, indicating inhibition of cell proliferation (*P < 0.05). Results in (a), (c), and (d) show the means ± S.D. of three independent experiments.

Using the CCK-8 assay, we found that the optimal concentration and time for cyclopamine treatment of cultured FLS were 10**μ**mol/L and 48 hours, respectively (data not shown). Results showed that cell proliferation rates in the cyclopamine treated group (72.5 ± 6.67)% were lower than those in the control group (100 ± 0)% (*P* < 0.05) (Figure 7(a)).

The effect of cyclopamine on cell cycle of cultured FLS from RA was observed by flow cytometry (Table 2 and Figures 7(c) and 7(d)). Results showed that cyclopamine reduced the percentage of cells in S + G_2_ phase (*P* < 0.05) and contributed to cell accumulation in G_1_ phase. (*P* < 0.05).

## 4. Discussion

As a morphogen, Shh is essential in pattern formation and in the regulation of stem cell and progenitor cell proliferation during embryogenesis and in adult tissues, and it also mediates angiogenesis under pathological conditions [15–18]. Smo is believed to be a positive regulator of Shh signaling and to be essential for pathway activation, either through ligand-dependent or ligand-independent mechanisms [19]. Excessive proliferation of FLS and synovial angiogenesis has a major role in initiating and perpetuating RA synovitis and joint destruction. However, the role of the Shh signaling pathway in FLS proliferation in RA has not been studied. Our results show that Shh, Ptch1, Smo, and Gli1 proteins were highly expressed in synovial tissue of patients with RA, especially in FLS, while expression in synovial tissue of patients with knee trauma was much lower. Based on these findings, we hypothesize that Shh signaling pathway activation may play an important role in excessive proliferation of FLS in RA synovium.

In general, it is accepted that enhanced Shh signaling pathway activation leads to downstream expression of target genes, including Ptch and Gli1, and therefore the levels of these transcripts are often used as surrogate markers of Shh pathway activity [20]. Using real-time PCR, immunofluorescence, and western blotting, we found that cultured FLS from RA patients expressed Shh pathway related components, such as Shh, Ptch1, Gli1, and Smo, and activated Shh transcriptional targets both at mRNA and protein levels. These results indicated that the Shh signaling pathway was activated in cultured RA FLS even after serum starvation, and cultured FLS from RA patients could be used as a cell model to investigate the role of the Shh signaling pathway in the excessive proliferation of FLS.

Cyclopamine is a naturally occurring alkaloid of the corn lily* Veratrum californicum*. It blocks the hedgehog signaling pathway by directly binding to Smo and inducing a conformational change similar to that induced by Ptch1. It is believed to be a specific antagonist of the hedgehog signaling pathway because it induces a developmental phenotype similar to that induced by disruption of hedgehog ligand and therefore could consequently silence the Shh signaling pathway [21].

Whether cyclopamine could suppress the activation of the Shh signaling pathway in cultured RA FLS was still unknown. Using cultured FLS from RA patients as a model, our experimental data showed that relative expression levels of Shh, Ptch1, Gli1, and Smo mRNA in the cyclopamine treated group were lower than those in the control group* in vitro*. Consistent with mRNA levels, western blot analysis also showed that protein expression levels of Shh and Smo in the cyclopamine treated group were lower than those in the control group* in vitro*. The results indicated that, similar to carcinoma cells, activation of the Shh signaling pathway in cultured RA FLS can be blocked by cyclopamine.

Recent studies have shown that blockage of the Shh signaling pathway by pharmacologically targeting the Smo receptor caused regression of tumor vasculature and inhibition of tumor growth [22–24]. Whether cyclopamine can inhibit cell viability and proliferation of RA FLS is still unknown. A previous study [25] showed that cyclopamine reduced Shh pathway activity in a dose-dependent manner, with a minimum of 5**μ**mol/L required to inhibit signaling significantly. In this study, cell viability and proliferation were observed using the CCK-8 assay. The results show that cyclopamine inhibited cell proliferation in a time- and concentration-dependent manner, with the optimal time and dose being 48 hours and 10**μ**mol/L. Flow cytometry analysis was then performed to further observe the effects of cyclopamine on cell cycle. The data showed that the percentage of cells in the G_1_ phase was increased, while the percentage of cells in G_2_ phase and S phase was decreased in cyclopamine-treated FLS. This indicated possible cell cycle arrest in the G_1_ phase and inhibition of entry into the S phase by cyclopamine. These results therefore support that both cell viability and proliferation were inhibited by cyclopamine.

## 5. Conclusion

Our studies revealed that the Shh signaling pathway is activated in joint synovium of RA patients* in vivo* and in cultured RA FLS* in vitro*. It may therefore play an important role in the proliferation of RA FLS, suggesting a new therapeutic approach to RA, which warrants further investigation.

## Figures and Tables

**Table 1:** Semi-quantitative analysis of Shh, Ptch1, Smo and Gli1 protein expression.

Group	Shh				Ptch1				Smo				Gli1			
	−	+	++	+++	−	+	++	+++	−	+	++	+++	−	+	++	+++
RA (*n* = 10)	0	2	8	0	0	6	4	0	0	0	6	4	0	8	2	0
Control (*n* = 5)	0	3	2	0	0	3	2	0	3	2	0	0	0	5	0	0

The positive expression rate of Shh signal pathway components (Shh, Ptch1, Smo and Gli1) in RA synovial tissues was examined by IHC. Results showed that the rate of moderately positive expression of Shh, Smo and Gli1 in the RA group (80%, 60% and 20%, resp.) was higher than in the control group (40%, 0% and 0%, resp.).

**Table 2:** Effect of cyclopamine on the cell cycle of cultured FLS from RA patients using flow cytometry.

Group	G_1_ phase (%)	G_2_ phase (%)	S phase (%)	(S + G_2_) phase (%)
Control (*n* = 3)	73.8 ± 1.05	11.1 ± 3.15	15.08 ± 3.97	26.22 ± 0.21
Cyclopamine group (*n* = 3)	92.76 ± 1.74^#^	0 ± 0	7.24 ± 1.74	8.05 ± 1.70^#^

Cell proliferation was assessed using flow cytometry. The results show the means ± S.D. of 3 independent experiments. The percentage of cyclopamine-treated FLS in the G_1_ phase was higher than that in the control group, whereas the percentage of cyclopamine-treated FLS in the S + G_2_ phases was lower than that in the control group.

^#^ *P* < 0.05 versus control group.
